# Comprehending unwanted hysterectomies in developing nations: an analysis of women’s decision-making processes, underlying causes, and subsequent consequences

**DOI:** 10.1097/MS9.0000000000002642

**Published:** 2024-10-08

**Authors:** Shreya Khandelwal

**Affiliations:** B.P. Koirala Institute of Health Sciences, Dharan, Nepal

## Introduction

The excessive prevalence of unnecessary hysterectomies presents a pressing and urgent challenge within healthcare systems globally, with particularly severe manifestations in the Southern regions of India. Data reveals an alarmingly high rate of hysterectomies in states such as Andhra Pradesh (8.7%) and Telangana (8.2%), compared to significantly lower rates in the Northeastern region (1.2%). Notably, the majority of these procedures occur in the private sector (69.6%), though a higher proportion is performed in public health facilities in the Northeastern region (73%) compared to private facilities (26.7%)^1^.

This research aims to uncover the underlying causes of this troubling trend, including the financial incentives that may prompt healthcare providers to recommend hysterectomies and the prevalent lack of awareness regarding alternative treatments for gynecological conditions. The consequences of unnecessary hysterectomies are profound, impacting individual health outcomes and contributing to broader systemic issues within healthcare. These procedures can lead to premature menopause, loss of fertility, and long-term hormonal imbalances^2^.

The issue extends beyond India, with similar patterns observed in Africa, Latin America, Eastern Europe, and the Middle East. For instance, private healthcare systems in Latin America often incentivize hysterectomies through higher reimbursement rates^2^. In Germany, data indicates that the peak age for hysterectomies aligns with earlier peaks observed in the United States, Denmark, and Brazil, specifically among women aged 45–49 years^3–6^. Even in developed countries like the United States, financial incentives and defensive medical practices contribute significantly to elevated hysterectomy rates^2^.

By exploring these factors and comparing hysterectomy practices across regions, this study aims to illuminate the complex dynamics influencing the overuse of hysterectomies in Northern India. Our goal is to raise awareness about this issue, advocate for more informed decision-making processes, and contribute to the broader discourse on women’s reproductive health and healthcare practices. Through this research, we seek to promote better health outcomes for women and drive meaningful improvements in healthcare delivery.

## Reasons behind the high rate of unwanted hysterectomies

The high rate of unwanted hysterectomies in India is influenced by multiple factors. A prevailing belief among women that the uterus is a burdensome organ contributes to the willingness to undergo the procedure without genuine medical necessity^7^. Economic constraints further exacerbate the issue, as gynecological treatments are often perceived as costly, particularly for economically disadvantaged women who may view hysterectomy as a cost-effective, one-time solution^7^. Additionally, inadequate access to primary healthcare services and misconceptions about hysterectomy as a comprehensive fix contribute to its prevalence, especially among women not using contraception and seeking a permanent solution for their health issues^7^.

Misinformation and fear of cancer, coupled with misdiagnosis of conditions like pelvic pain as pelvic inflammatory disease, drive women toward unnecessary hysterectomies^7^. The combination of societal beliefs, economic factors, lack of healthcare access, and misinformation highlights the need for comprehensive education and awareness campaigns to address this concerning trend.

## Prevalence of unwanted hysterectomies in other parts of the world

Unwanted hysterectomies, performed without genuine medical necessity, are a global issue spanning Africa, Latin America, Eastern Europe, and the Middle East. These practices are driven by a mix of financial incentives for healthcare providers, entrenched societal norms, economic constraints, and limited access to alternative treatments. In rural Africa, insufficient healthcare infrastructure contributes to high rates of unnecessary surgeries. In Latin America, private healthcare systems incentivize hysterectomies through elevated reimbursement rates. Outdated medical practices in Eastern Europe and societal norms in the Middle East also play roles in the prevalence of unnecessary hysterectomies^7^.

In the United States, approximately one in five women, and half of those aged 70 years or older, report having undergone a hysterectomy. Data from a large population-based sample reveals that hysterectomy prevalence peaks among women aged 70–79 years (46.7%) and those aged 80 years and older (48.8%). Higher prevalence is also noted among non-Hispanic Black women (21.3%), non-Hispanic American Indian and Alaska Native women (21.1%), and those residing in the South (21.1%)^2^. Despite a modest decline in prevalence from 18.9% in 2012 to 17.0% in 2020, the persistently high rates among certain demographic groups highlight the need for continued scrutiny and reform. Addressing underlying factors such as financial incentives, outdated medical practices, and sociocultural biases is crucial for reducing unnecessary hysterectomies and improving patient outcomes.

## Financial incentives for doctors to perform hysterectomies

Financial incentives significantly influence the prevalence of hysterectomies, particularly in specific regions and healthcare settings. Government-provided monetary rewards for procedures like hysterectomies under national rural health missions have led to increased rates of these surgeries, especially in private hospitals in urban areas^8^. This financial motivation creates a scenario where some doctors may exploit disadvantaged and less educated women to generate quick income, leading to unethical practices and unnecessary procedures^8,9^. Unscrupulous healthcare providers and private clinics often exploit government health schemes by claiming inflated insurance fees for hysterectomies, exacerbating the rates of these surgeries among certain demographics^8,9^. This situation underscores the need for stricter oversight and regulations to ensure that hysterectomies are performed only when medically necessary and in the best interest of the patient.

## Lack of awareness about alternative treatments for gynecological issues

The lack of awareness about alternative treatments for gynecological issues perpetuates a cycle of misinformation and unnecessary medical interventions. In India, private medical care has been criticized for overmarketing hysterectomy as a one-size-fits-all solution, often without considering alternative treatments^8^. This lack of informed consent is particularly concerning among rural, illiterate women who may undergo surgeries without understanding the potential risks and consequences^7^. Without awareness of alternative treatments, both women and healthcare providers may resort to hysterectomy as a permanent solution, overlooking less invasive options^7,10^. The repercussions of this lack of awareness include unnecessary medical expenses, physical burdens, and detrimental health outcomes, such as surgical complications, hormonal imbalances, osteoporosis, and cardiovascular disease. Psychologically, women may experience emotional distress, loss of femininity, and changes in body image post-surgery. Socioeconomically, hysterectomy can increase medical expenses and reduce productivity, particularly for women in developing countries where financial stability is precarious^7^. Additionally, societal pressures from employers to maintain productivity can coerce women into invasive surgeries, highlighting the broader implications of insufficient knowledge about alternative treatments^11^.

## Conclusion

This research paper investigates the pressing issue of unwanted hysterectomies in India and other parts of the world, examining the complex interplay of societal, economic, and healthcare-related factors that contribute to this widespread practice. The findings reveal that pressures from employers, the perception of hysterectomy as a cost-effective solution, and misinformation contribute to the high rate of unnecessary surgeries. Financial incentives in healthcare settings further exacerbate the problem, indicating a systemic issue requiring comprehensive intervention. The study also underscores the need for education and awareness campaigns to challenge deep-rooted societal beliefs and improve diagnostic practices. Addressing these issues through enhanced education, improved healthcare access, and patient-centered care is essential to mitigating the adverse impacts of unwanted hysterectomies on women’s health and well-being.

## Ethical approval

This editorial adheres to the principles. The collection and assessment of all confidential patient health data were conducted in compliance with the Health Insurance Portability and Accountability Act (HIPAA).

## Consent

No consent was required for this editorial.

## Source of funding

The author(s) received no financial support for the research, authorship and/or publication of this case report.

## Author contribution

The author has contributed substantially to all aspects of the editorial as the main author, including study concept and design, data collection, and writing of the paper.

## Conflicts of interest disclosure

The authors declare that they have no conflicts of interest.

## Research registration unique identifying number (UIN)

Not required for this editorial.

## Guarantor

No guarantor was involved.

## Data availability statement

No new data were created or analyzed in this study. Data sharing is not applicable to this article.

## Provenance and peer review

Not commissioned, externally peer-reviewed.
